# Genome editing in insects: current status and challenges

**DOI:** 10.1093/nsr/nwz008

**Published:** 2019-02-05

**Authors:** Jun Xu, Xia Xu, Shuai Zhan, Yongping Huang

**Affiliations:** CAS Key Laboratory of Insect Developmental and Evolutionary Biology, CAS Center for Excellence in Molecular Plant Sciences, Shanghai Institute of Plant Physiology and Ecology, Chinese Academy of Sciences, China

Insects constitute the largest group in the animal kingdom, and include many economically important species as well as pests. This abundance provides large-scale genetic resources, and research on insects is beneficial from the perspectives of human health, agricultural production and basic science (Fig. 1).

**Figure 1.**
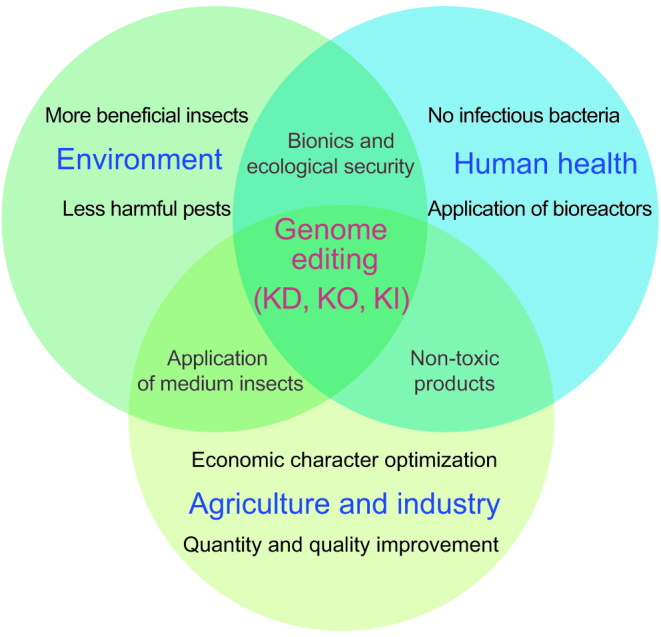
Genome editing has broad applications in insects. There are extensive effects on the environment, human health, industry and agriculture. It can realize the utilization of beneficial insects and pest control in the environment. It also can reduce the spread of bacteria and promote the development of bioreactors. Moreover, it provides high-quality raw materials for industry and agriculture, so as to improve their quantity and quality.

The availability of many genetic tools has made *Drosophila melanogaster* a crucially important model organism for basic biology research. However, many interesting biological hallmarks lie in non-*Drosophila* species, in which gene manipulation and related *in vivo* systems are still difficult. The recent establishment of genome editing techniques provides the capacity for gene knock-out (KO), knock-in (KI), and/or knock-down (KD) in non-model systems. Two types of genome editing tools, including transcriptional activator-like effector nucleases (TALENs) and clustered regularly interspaced short palindromic repeats (CRISPR)/Cas9, are available and are frequently applied. The CRISPR/Cas9 system involves the invariable Cas9 protein and easily designed single-guide RNAs (sgRNAs), while TALEN needs to be redesigned to target different loci each time. Thus, the CRISPR/Cas9 is technically simple and more widely used.

## PROGRESS IN INSECT GENOME EDITING

Although scientists have exploited a series of substantial genetic tools using *Drosophila* as a model insect, genome editing brings great convenience and reliability to gene function research in *Drosophila*. For example, CRISPR-based screening has lower off-target effects than RNA interference (RNAi) [1]. The precise site insertion induced by CRISPR-based KI promotes tag-based protein/cell studies for protein localization, protein interaction, cell lineage development and localization [2]. KI can avoid the side effects of transgenes, and demonstrate the true transcription and translation of genes or proteins in a cell [3].

Gene editing tools can be used to generate novel economic insect strains more rapidly and with greater efficiency than traditional breeding methods [4]. Chen *et al*. used transgenic CRISPR/Cas9-mediated KO of nuclear polyhedrosis virus (NPV) genes to develop a new silkworm strain with a high degree of resistance to *Bombyx mori* nuclear polyhedrovirus infection [5]. Using TALEN-mediated homology-directed repair (HDR), Xu *et al*. replaced the silkworm fibroin heavy chain gene (*FibH*) with the major ampullate spidroin-1 gene (*MaSp1*) from the spider *Nephila clavipes* [6]. This editing method also yielded a male-only strain in which the sex ratio of silkworms could be regulated through W chromosome insertion. A cassette with an embryonic lethal gene KO function can be inserted into a fragment on the W chromosome resulting in female lethality. This method is not only useful for the silk industry, as males produce silk of higher quality and of a greater quantity than females, but will also be helpful for the development of environmentally friendly pest control strategies for lepidopteran pests [7].

Gene drive targets with functional or structural constraints that might prevent the development of resistant variants could offer a route to successful vector or pest population control. Bier and colleagues have established a new CRISPR/Cas9-based method, called ‘active genetics’ that can greatly bias the transmission of genetic traits, thereby bypassing the traditional constraints of Mendelian inheritance [8]. This new technology has been applied to develop gene drive systems to target *doublesex*, causing complete population suppression in malaria vector *Anopheles gambiae* mosquito populations and all-male population collapse of the agricultural pest *Ceratitis capitata* [8,9]. These numerous benefits have established genome editing methods as important tools.

Genome editing provides an easy and effective approach to generate loss-of-function mutants for functional genetic studies. CRISPR/Cas9 has provided answers regarding wing color mimicry of butterflies and social behavior in ants [10,11].

## CHALLENGES OF GENOME EDITING IN INSECTS

### Delivery technology

Despite a large amount of research effort, genome editing tools have still only been applied to a limited number of insect species, including the silkworm, butterflies, a few moths, mosquitoes and locusts. This is due to the difficulty of applying microinjection techniques in insect eggs. Many parameters need to be explored and optimized in each species, including (but not limited to) appropriate penetration techniques, egg collection time, injection timing, injection placement, the method for sealing eggs after injection and incubation conditions. The embryos of many insects have a hard shell, which makes microinjection difficult. For example, a double-needle system, in which a tungsten needle pierces the egg shell and a capillary glass needle injects plasmids, is used for microinjection in the silkworm. One group has developed a technology termed Receptor-Mediated Ovary Transduction of Cargo to deliver Cas9 ribonucleoprotein (RNP) to the arthropod germline by injection into adult female mosquitoes without injecting eggs [12].

### Screening

The major obstacle is that it is not easy to select an edited insect from the brood population. Such screening problems can be resolved using transgenic-based genome editing with select marker genes, such as fluorescent protein genes or body color genes. One method involves the use of the ribozyme-gRNA-ribozyme (RGR) structure with multiple sgRNAs—some for the target genes and others for genes involved in body color—in a single vector, thus allowing selection of mutants based on readily detectable visual characteristics (i.e. fluorescence or body color).

### KI efficiency

CRISPR-based KI has failed many times in the silkworm and other lepidopteran insects. Compared with successful TALEN-based KI, HDR has a higher probability of occurrence than non-homologous end joining (NHEJ), which induces DNA double-strand breaks (DSBs) in CRISPR/Cas9. Inhibition of the NHEJ pathway factors Ku70 and DNA ligase 4 can improve KI efficiency [13]. The endonuclease *Cpf*1 has been reported to produce cohesive ends after DSBs that are suitable for HDR repair [14].

### Off/on-target effects

Although the probability of off-target effects with CRISPR/Cas9 is much lower than it is with RNAi, the potential occurrence of such effects also leads to experimental inaccuracies. Computer-aided sgRNA design (CRISPRdirect: https://crispr.dbcls.jp/) can reduce the likelihood of off-target effects [15]. In some cases, several target sgRNAs may be required to edit a single gene because the DNA sequences may not break.

In entomological research, especially in non-*Drosophila* insects, future work should focus on the development of precise genome editing methodologies, with high efficiency and low probabilities of off-target effects for large-fragment KI and single-base pair editing. It will also be necessary for RNA function to be studied and novel techniques to be developed to edit non-coding RNA, piwi-interacting RNA, microRNAs and so on. Additionally, due to their ecological importance, the effects of transferring gene-edited insects from the laboratory to the wild will require further study, monitoring and the development of specific regulations.
